# The Contractile Function of Ventricular Cardiomyocytes Is More Sensitive to Acute 17β-Estradiol Treatment Compared to Atrial Cardiomyocytes

**DOI:** 10.3390/cells14080561

**Published:** 2025-04-08

**Authors:** Tatiana A. Myachina, Xenia A. Butova, Raisa A. Simonova, Denis A. Volzhaninov, Anastasia M. Kochurova, Galina V. Kopylova, Daniil V. Shchepkin, Anastasia D. Khokhlova

**Affiliations:** 1 Institute of Immunology and Physiology UrB RAS, 620049 Yekaterinburg, Russia; myachina.93@mail.ru (T.A.M.); raisa.simonova@mail.ru (R.A.S.); volzhaninovdenis@yandex.ru (D.A.V.); cmybp@mail.ru (D.V.S.); 2 Institute of Natural Sciences and Mathematics, Ural Federal University, 620026 Yekaterinburg, Russia; 3 Department of Biomedical Engineering, Washington University, St. Louis, MO 63130, USA; azelgadiss@yandex.ru

**Keywords:** 17β-estradiol, atria, ventricles, single cardiomyocyte, sarcomere shortening-relengthening, tension–length relationship, actin–myosin interaction, contractile protein phosphorylation

## Abstract

17β-estradiol (E2) is the most active metabolite of estrogen with a wide range of physiological action on cardiac muscle. Previous studies have reported E2 effects predominantly for the ventricles, while the E2 impact on the atria has been less examined. In this study, we focused on the direct E2 effects on atrial and ventricular contractility at the cellular and molecular levels. Single atrial and ventricular cardiomyocytes (CM) from adult (24 weeks-old) female Wistar rats were incubated with 10 nM E2 for 15 min. Sarcomere length and cytosolic [Ca^2+^]_i_ transients were measured in mechanically non-loaded CM, and the tension–length relationship was studied in CM mechanically loaded by carbon fibers. The actin–myosin interaction and sarcomeric protein phosphorylation were analyzed using an in vitro motility assay and gel electrophoresis with Pro-Q Diamond phosphoprotein stain. E2 had chamber-specific effects on the contractile function of CM with a pronounced influence on ventricular CM. The characteristics of [Ca^2+^]_i_ transients did not change in both atrial and ventricular CM. However, in ventricular CM, E2 reduced the amplitude and maximum velocity of sarcomere shortening and decreased the slope of the passive tension–length relationship that was associated with increased TnI and cMyBP-C phosphorylation. E2 treatment accelerated the cross-bridge cycle of both atrial and ventricular myosin that was associated with increased phosphorylation of the myosin essential light chain. This study shows that E2 impairs the mechanical function of the ventricular myocardium while atrial contractility remains mostly preserved. Hormonal replacement therapy (HRT) with estrogen is by far the most effective therapy for treating climacteric symptoms experienced during menopause. Here we found a chamber specificity of myocardial contractile function to E2 that should be taken into account for the potential side effects of HRT.

## 1. Introduction

17β-estradiol (E2), the most biologically active form of estrogen, is produced by the gonads, muscles, and adipose tissue due to the expression of aromatase (P450_arom_), which converts androgens to estrogens [1]. It has been shown that myocardial tissue levels of E2 are comparable with E2 plasma levels in rats [2]. Three main E2 receptors, ERα, ERβ, and GPR30 (also known as GPER1), are present in cardiomyocytes (CM) and allow for direct E2 signaling within the heart tissue. E2 plays an important role in the development and function of the myocardium through various mechanisms, such as protein synthesis, signaling, autophagy, the cellular antioxidant system, etc. [3,4,5,6], that influence the phosphorylation of sarcomere proteins and the Ca^2+^ cycle in CM through activation of NO production [5,7].

The data on the E2 effects on myocardial contractility and structure are contradictory and depend on the experimental model and E2 concentrations used. Most works showed that gonadal E2 deficiency caused left ventricular (LV) hypertrophy, decreased myocardial vascularization, collagen deposition, impaired Ca^2+^ signaling, and myocardial contractility [7,8,9,10,11,12,13,14]. However, some findings demonstrated that short- or long-ovariectomy did not induce LV remodeling [15] or resulted in an increase in the amplitudes of fractional shortening and cytosolic Ca^2+^ concentration ([Ca^2+^]_i_) transients in intact ventricular CM [12] and elevated force of skinned ventricular CM [9]. The latter is consistent with an increased amplitude of CM shortening observed under E2 deficiency in a genetic model of aromatase tissue deficiency in female mice [16].

Hormonal replacement therapy (HRT) with E2 to ovariectomized animals corrected CM contractile dysfunction elicited by ovariectomy [10]. E2 prevented an increase in LV mass and the ejection fraction reduction but did not prevent a decrease in LV shortening fraction [17]. E2 has been shown to have cardioprotective effects in many studies in various models of cardiac diseases. The incubation of cultured rat right ventricular CM with E2 restored CM contractility and cytosolic Ca^2+^ levels, which were decreased in the monocrotaline model of pulmonary hypertension [18]. E2 treatment in ovariectomized mice reduced ventricular hypertrophy and attenuated deterioration in ventricular contractility after transverse aortic constriction [8], significantly decreased mortality, and reduced infarct size and CM apoptosis after myocardial infarction [19]. On the other hand, some studies have found that E2 at high concentrations causes negative inotropic effect with impaired contractility and Ca^2+^ handling in CM [20,21,22] and promotes myocardial hypertrophy [23] and overexpression of aromatase [24] in the myocardium.

Several studies reveal that E2 also contributes to arrhythmogenicity of the ventricular and atrial myocardium. In the Langendorff-perfused guinea pig hearts and single ventricular CM, E2 at its physiological concentration acutely affects the potassium channel gating, action potential duration (APD), and QT prolongation [25]. Mice with overexpression of aromatase in the myocardium were sensitive to myocardial ischemia-reperfusion, showing an increased duration of ventricular fibrillation and a longer period of contractile activity recovery after coronary occlusion [24]. At the same time, E2 can decrease reperfusion arrhythmias in multiple animal studies, particularly through the upregulation of ERβ [26,27,28]. While much research has focused on the effects of E2 on ventricular function and arrhythmias, less is known about its specific impact on atrial sensitivity. HRT containing estrogen, except estradiol-only HRT, increased the risk of atrial fibrillation in patients [29]. In female mice, E2 application delayed atrial conduction, resulted in heterogeneity in APD, and caused atrial arrhythmias [30,31].

The differing sensitivity of atria and ventricles to E2 treatment underscores the complexity of estrogen effects on cardiac function. Previously, we have shown that in ovariectomized rats, the force production is decreased in LV single CM but increased in left atrial CM, which can be associated with different sensitivity of atrial and ventricular contractile proteins to E2 deficiency [32]. The expression of estrogen receptors differs between atria and ventricles, which may provide different E2 influences on the atrial and ventricular myocardium, potentially affecting cardiac function and disease susceptibility [33]. The aim of this study is to assess the direct effects of E2 on the contractile function of atrial and ventricular myocardium using freshly isolated rat CM and contractile proteins. We have assessed the E2 effects on sarcomere length dynamics and [Ca^2+^]_i_ transients in mechanically non-loaded single cardiomyocytes, force production and tension–length relationship in mechanically loaded cardiomyocytes, and sarcomere protein function.

## 2. Materials and Methods

### 2.1. Animals and Ethical Approval

All experiments were performed according to Directive 2010/63/EU of the European Parliament (NIH Publication No. 85–23, revised 1985) and approved by the Animal Care and Use Committee of the Institute of Immunology and Physiology of RAS (protocol No. 03/21 from 1 December 2021). Female 24-week-old Wistar rats were obtained from the animal house of the Institute of Immunology and Physiology. The animals were kept in the same conditions: caged separately in groups of 5–6 per cage in a room at 22–24 °C under a 12:12 h light-dark cycle and with unlimited access to food (Delta Feeds LbK 120 S-19, BioPro, Novosibirsk, Russia) and water. For the experiments, rats were deeply anesthetized with Zoletil-100 (Virbac, Westlake, TX, USA) at a dose of 0.3 mL/kg with 2% Xylazine (Alfasan, Woerden, The Netherlands) at a dose of 1 mL/kg. Unless otherwise noted, all chemicals and reagents were purchased from Merck (St. Louis, MO, USA).

### 2.2. Experimental Design

Here we used 10 nM of E2, which is above physiologically relevant systemic serum concentrations of E2 in the range of 0.01–1.0 nM for female rats [34]. In pregnant women, E2 concentrations increase to several hundred nM [25]. It was shown that 10 nM of E2 unlikely may cause acute toxicity [35]. A dose of 10 nM estradiol is commonly used in in vitro studies to investigate the direct cellular responses, including effects on proliferation, mitochondrial function, and contractility [36,37]. 17β-estradiol (E8875, Sigma-Aldrich, St. Louis, MO, USA, E2) was dissolved in 96% ethanol as a stock solution of 1 μM according to the manufacturer’s instructions and stored at 2–4 °C. On the day of the experiment, the stock solution was diluted in a HEPES-buffered Tyrode solution (in mM: 140 NaCl, 5.4 KCl, 1.0 MgSO_4_, 10 HEPES, and 11.1 glucose, and 1.8 CaCl_2_, pH 7.35 with NaOH) to 10 nM. Single atrial and ventricular CM were pretreated with 10 nM E2 or ethanol (vehicle control) in Tyrode’s solution (36 ± 1 °C) for 10 min before measurements. Measurements were performed within 5 min immediately after the incubation. CM from the control group were incubated in a Tyrode solution with ethanol (the final concentration in a solution was 0.0005%) following the same timeline as the E2 group. To study the actin–myosin interaction and sarcomeric protein phosphorylation, CM suspensions were incubated as for single-cell studies, and then cardiac myosin and protein samples for gel staining were prepared.

### 2.3. Isolation of Single CM from the Atria and Ventricles

Single CM from atria and ventricles were obtained according to a combining method of Langendorff—the perfusion and injection techniques are described in detail elsewhere [38,39]. In brief, animals were intramuscularly injected with 5000 IU/kg sodium heparin (Ellara, Pokrov, Russia) before the heart isolation. The heart was cannulated via the aorta and perfused at a rate of 4.0–4.5 mL/min at 35.5 °C using the Langendorff apparatus with a physiological solution (in mM: 140.0 NaCl, 5.4 KCl, 1.2 MgSO_4_, 10.0 HEPES, 20.0 taurine, 5.0 adenosine, 11.1 D-glucose, 1.0 CaCl_2_, pH 7.35 with NaOH) for 5 min. Then, the solution was changed to a low-Ca^2+^-high K^+^ solution (in mM: 115.0 NaCl, 14.0 KCl, 1.2 MgSO_4_, 10.0 HEPES, 20.0 taurine, 5.0 adenosine, 11.1 D-glucose, 0.3 EGTA, 0.025 CaCl_2_, pH 7.15 with NaOH) for 15 min. Afterward, the heart was enzymatically digested with an EGTA-free-high K^+^ enzyme solution containing 0.8 mg/mL collagenase II (~305 IU/mL; Worthington, Biochemical, Lakewood, NJ, USA) and 0.06 mg/mL protease XIV (~3.5 IU/mL). During the Langendorff perfusion with an enzyme solution, atria were injected with an EGTA-free-high K^+^ enzyme solution containing 1.0 mg/mL collagenase II and 0.06 mg/mL protease XIV. After 10–15 min, the heart was transferred to a Petri dish for direct injections into the cardiac chambers with an enzyme solution (0.9 mg/mL collagenase II and 0.06 mg/mL protease XIV) for ≈25 min. Atria and ventricles were separated, cut, and re-suspended with an EGTA-free-high K^+^ solution supplemented with bovine serum albumin (5 mg/mL). Finally, extracellular Ca^2+^ concentration (0.1–1.8 mM) was gradually adjusted. Isolated cardiomyocytes were stored at room temperature in a HEPES-buffered Tyrode solution with 1.8 mM CaCl_2_. Experiments were performed after allowing CMs to rest for at least 30 min.

### 2.4. Measurements of Sarcomere Length Dynamics

Sarcomere length (SL) dynamics during mechanically non-loaded CM contractions were recorded using a fast Fourier transformation-based algorithm using the IonOptix system and Ion Wizard software 6.6 (IonOptix Corporation, Milton, MA, USA). Measurements were performed at a stimulation frequency of 1 Hz at 36 ± 1°C. The last ten steady-state contractions were averaged, and the following parameters were analyzed: end-diastolic sarcomere length (EDSL), absolute sarcomere shortening amplitude (EDSL minus end-systolic SL), fractional sarcomere shortening amplitude (absolute sarcomere shortening amplitude normalized by EDSL, FS), maximum velocities of sarcomere shortening (SL v_short_) and relengthening (SL v_rel_), time-to-peak sarcomere shortening (SL TTP), and time to 50% sarcomere relengthening (SL TTR_50_).

### 2.5. Measurements of Cytosolic [Ca^2+^]_i_ Transients

Cytosolic [Ca^2+^]_i_ transients were recorded in a narrow region of mechanically non-loaded CM at 1 Hz and 36 ± 1 °C using a laser scanning microscopic system LSM 710 and Zen 2008 software (Carl Zeiss, Jena, Germany). CM were incubated with 1.7 µM Fluo-8AM fluorescent dye (AAT Bioquest, Sunnyvale, CA, USA) and 0.1% Pluronic^®^ F-127 (AAT Bioquest, Sunnyvale, CA, USA) in darkness for 20 min at room temperature followed by washing with a Tyrode solution. The Fluo-8AM was excited using an Ar-laser at 488 nm, and the fluorescence was emitted at 493–575 nm. The changes in the fluorescence signal were calculated as ΔF/F_0_, where F_0_ is the minimal fluorescence intensity measured between contractions at the diastolic phase of [Ca^2+^]_i_ transients. The following parameters of cytosolic [Ca^2+^]_i_ transients were analyzed using custom-made software EqapAll 6.0 [40]: an amplitude of [Ca^2+^]_i_ transients ([Ca^2+^]_i_ Ampl), time-to-peak [Ca^2+^]_i_ transients ([Ca^2+^]_i_ TTP), and time to 50% [Ca^2+^]_i_ decay ([Ca^2+^]_i_ TTD50).

### 2.6. Measurements of Tension-Length Dependence in a Single Cardiomyocyte

Measurements of the CM tension–length relationship were performed using a carbon fiber technique as described in detail elsewhere [41]. In brief, four carbon fibers (≈10 µm in diameter, Tsukuba Materials Information Laboratory, Tsukuba, Japan) were electrostatically attached to the top and bottom surfaces of the CM on the left and right cell edges. Each carbon fiber was connected to a digital micromanipulator (Sensapex, Oulu, Finland) for precise positioning. The left pair carbon fiber rigidly fixed the CM end, preventing it from moving. The right pair carbon fiber received the movement command to apply stretch (preload) on a CM using custom-made software [42].

During the tension-length protocol, auxotonically contracting CM under the carbon fiber load were stretched by ≈5% from the initial EDSL every 20–30 contraction cycles (with a step of 2 µm per 300 ms), and for each stretch step, the last ten steady-state contractions were averaged and analyzed. The distance between the left and right carbon fiber tips (effective CM length) was measured optically using Ion Wizard software 6.6. Measurements were carried out at a stimulation frequency of 1 Hz and 30 ± 1 °C.

CM force was calculated as *F = K* (Δ*L*_*uMP*_ − Δ*L*_*CF*_), where K is the combined stiffness of right pair of carbon fibers (0.06–0.08 N × m^−1^) measured by a force transducer system (Aurora Scientific, ON, Canada), Δ*L_uMP_* is the change in micromanipulator position after stretch, and Δ*L_CF_* is the change in effective CM length. To obtain the CM tension, the CM force was normalized to the cell cross-sectional area calculated from the measured CM width, assuming an elliptical cross-section with a 3:1 ratio of long and short axes [43].

To compare tension-length dependence in CM between the groups, we analyzed the slopes of the end-diastolic (passive) tension–length relationship (EDTLR), end-systolic (total) tension–length relationship (ESTLR), and active tension–length relationship (ATLR). The magnitudes of passive, total, and active (total minus passive) tension were fitted by linear regression (coefficient of determination R^2^ > 0.9) against the end-diastolic effective cell length for EDTLR and end-systolic effective cell length for ESTLR with ATLR (cell length was expressed in % by initial cell length before the stretch). The Frank–Starling gain (FSG) index was calculated as ESTLR/EDTLR [44,45].

### 2.7. Studies of Actin–Myosin Interaction and Phosphorylation of Contractile Proteins

The functional properties of cardiac myosin were assessed by the estimation of the sliding velocity of F-actin over myosin in an in vitro motility assay. Cardiac myosin was extracted from the CM suspension according to Margossian and Lowey [46] with modifications. After incubation with E2, the CM suspension was centrifuged at 25,000× *g* for 15 min (Beckman Coulter Inc., Brea, CA, USA). To cell sediment, an equivalent volume of high-ion buffer (HIS buffer in mM: 1000 KCl, 40 phosphate buffer, 4 MgCl_2_, 10 DTT, 1 ATP, pH 6.5) was added, and this CM suspension was sonicated (Labsonic M, Sartorius AG, Göttingen, Germany). Sarcomeric proteins were extracted for 30 min, and CM were sedimented by centrifugation at 25,000× *g* during 15 min. The supernatant was used to determine the degree of phosphorylation of the main sarcomere proteins and to obtain myosin. To obtain myosin, the ionic strength of the solution was reduced 10-fold by adding deionized water for 30 min. Myosin was sedimented by centrifugation for 15 min at 25,000× *g* and dissolved with an equivalent volume of HIS buffer without ATP. F-actin was obtained from the pig left ventricles [47].

Phosphorylation of cardiac myosin-binding protein C (cMyBP-C), essential and regulatory light chain of myosin (ELC and RLC), troponin T and I (TnT and TnI), and tropomyosin (Tpm) was analyzed using a 12% SDS-PAGE with Pro-Q Diamond phosphoprotein (Invitrogen, Eugene, OR, USA) and SYPRO Ruby (Invitrogen, Eugene, OR, USA) staining. Protein samples and gel staining were prepared according to the manufacturer’s instructions. The gels were scanned with the ChemiDoc MP Imaging System (Bio-Rad, Hercules, CA, USA), and band densities were determined by Image Lab 5.2.1 software (Bio-Rad, Hercules, CA, USA). Protein phosphorylation was expressed as a ratio of the Pro-Q Diamond intensity to the SYPRO Ruby intensity.

### 2.8. Statistical Analysis

Analyzed characteristics were collected in Excel 16 (Microsoft Corp, Redmond, WA, USA) and processed by GraphPrism 10 (GraphPad Software, San Diego, CA, USA) for statistical analysis. The significance of E2 effects was assessed using the Mann–Witney U-test and considered when *p* < 0.05. Data are expressed as median and interquartile range using violin plots to demonstrate a whole range of values in groups.

## 3. Results

### 3.1. The Effects of 17β-Estradiol on Sarcomere Length Dynamics in Single Cardiomyocytes

**Figure 1:**
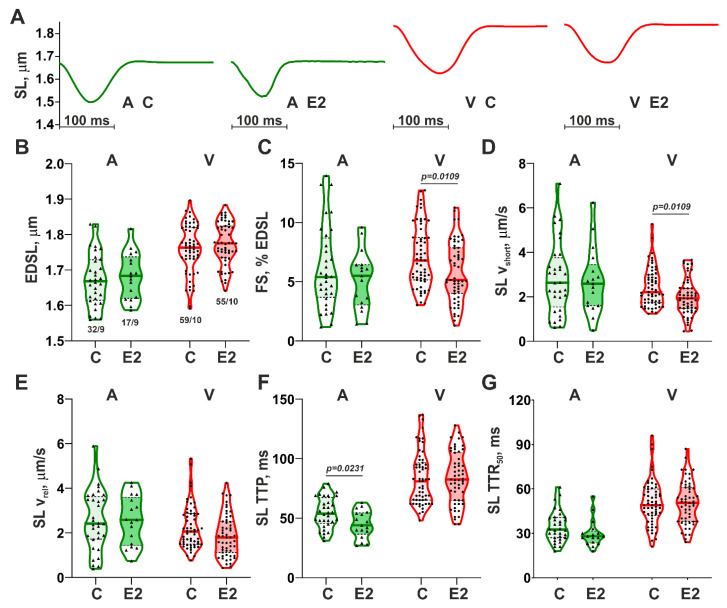
Effects of 17β-estradiol (E2) on sarcomere length (SL) dynamics in mechanically non-loaded contracting atrial and ventricular CM. (**A**) Representative traces of steady-state SL changes in atrial and ventricular CM from the control group (**C**) and after 15 min incubation with E2; (**B**) End-diastolic SL (EDSL); (**C**) Fractional sarcomere shortening (FS); (**D**) Maximum velocity of sarcomere shortening (SL v_short_); (**E**) Maximum velocity of sarcomere relengthening (SL v_rel_); (**F**) Time-to-peak sarcomere shortening (SL TTP); (**G**) Time to 50% sarcomere relengthening (SL TTR_50_). A—atrial CM; V—ventricular CM. Data are presented as violin plots: bold line shows median, dashed lines indicate an Q1–Q3 interval. n/N (number of CM from N hearts) is shown on the B panel. Each dot represents individual CM. The Mann–Whitney U-test is used to compare C and E2 groups.

First, we compared the E2 effects on the parameters of sarcomere length (SL) dynamics in mechanically non-loaded atrial and ventricular CM. Figure 1A shows the representative traces of steady-state SL changes in single CM from the control (vehicle) group (C) and after the 15 min incubation with E2. E2 application did not affect end-diastolic sarcomere length (EDSL) in atrial or ventricular CM (Figure 1B). In ventricular CM, E2 induced a decrease in both fractional sarcomere shortening (FS) and absolute sarcomere shortening amplitude by ~24% (Supplementary Materials, Figure S1) and reduced the maximum velocity of sarcomere shortening (SL v_short_) by ~12% (Figure 1C,D). In atrial CM, the application of E2 resulted in a ~18% decrease in time-to-peak sarcomere shortening (SL TTP, Figure 1F) without changes in other parameters of SL dynamics.

Thus, the effects of E2 on the SL dynamics are more pronounced in ventricular CM than in atrial CM, manifesting in the decreased amplitude and maximum velocity of sarcomere shortening. In atrial CM, E2 showed reduced time-to-peak sarcomere shortening, while the shortening amplitude was preserved.

### 3.2. The Effects of 17β-Estradiol on Cytosolic [Ca^2+^]_i_ Transients in Single Cardiomyocytes

**Figure 2:**
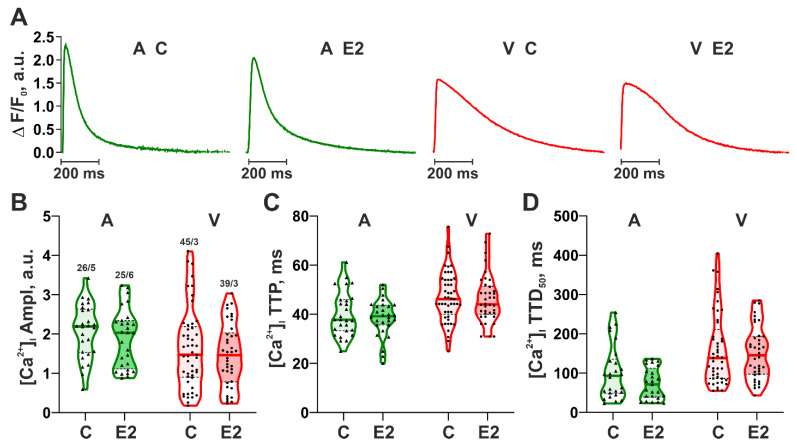
Effects of 17β-estradiol (E2) on cytosolic [Ca^2+^]_i_ transients in single mechanically non-loaded atrial and ventricular CM. (**A**) Representative traces of steady-state [Ca^2+^]_i_ transients in CM from the control group (C) and after 15 min incubation with E2; (**B**) An amplitude of [Ca^2+^]_i_ transients ([Ca^2+^]_i_ Ampl); (**C**) Time-to-peak [Ca^2+^]_i_ transients ([Ca^2+^]_i_ TTP); (**D**) Time to 50% [Ca^2+^]_i_ decay ([Ca^2+^]_i_ TTD_50_). A—atrial CM; V—ventricular CM. Data are presented as violin plots: bold line shows median, dashed lines indicate an Q1–Q3 interval. n/N (number of CM from N hearts) is shown on the B panel. Each dot represents an individual CM. The Mann–Whitney U-test is used to compare C and E2 groups.

Then, we analyzed the changes in cytosolic [Ca^2+^]_i_ as a main link of electro-mechanical coupling [48]. Incubation of mechanically non-loaded CM with E2 did not affect the amplitude and time-course parameters of the [Ca^2+^]_i_ transients in either ventricular or atrial CM (Figure 2). These data suggest that changes in SL dynamics induced by E2 are not associated with the E2 impact on the characteristics of [Ca^2+^]_i_ transients in CM.

### 3.3. The Effects of 17β-Estradiol on Tension-Length Dependence in Single Cardiomyocytes

**Figure 3:**
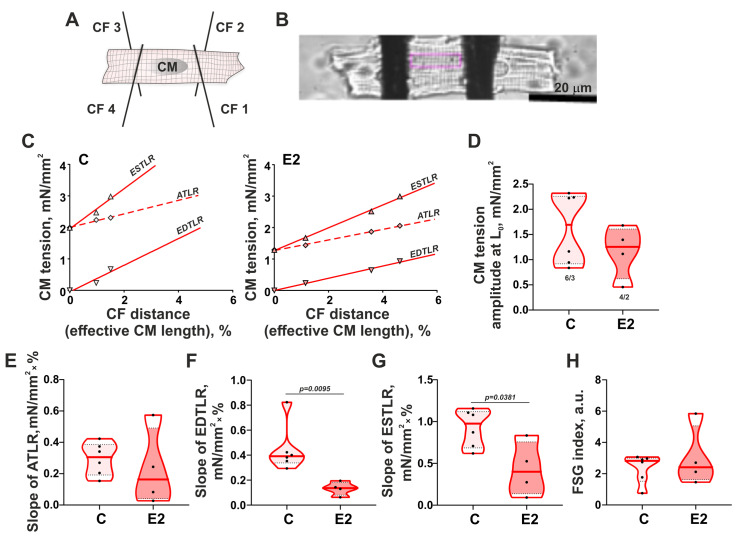
Effects of 17β-estradiol (E2) on the tension-length dependence in single ventricular CM. (**A**) Scheme of CM fixation by four carbon fibers (CF); (**B**) Representative photo of single ventricular CM attached to carbon fibers (top view); (**C**) Representative tension–length relationships in single CM from the control group (C) and after 15 min incubation with E2. To obtain the slopes of end-diastolic (passive) tension–length relationship (EDTLR), end-systolic (total) tension–length relationship (ESTLR), and active tension–length relationship (ATLR), the tension magnitudes were fitted by linear regression against the end-diastolic effective cell length for EDTLR and end-systolic effective cell length for ESTLR and ATLR (cell length was expressed in % by initial cell length before the stretch); (**D**) Tension amplitudes at the initial cell length before the stretch; (**E**) The slopes of ATLR. (**F**) The slopes of EDTLR; (**G**) The slopes of ESTLR. (**H**) Frank–Starling gain index (FSG) Frank–Starling gain (FSG) index calculated as ESTLR/EDTLR. Data are presented as violin plots: bold line shows median, dashed lines indicate an Q1–Q3 interval. n/N (number of CM from N hearts) is shown on the C panel. Each dot represents an individual CM. The Mann–Whitney U-test is used to compare C and E2 groups.

To verify that E2 induces contractility impairment of ventricular CM, a carbon fiber technique (Figure 3A,B) was used to mechanically load the CM and assess the tension-length dependence by applying the stretch protocol (Figure 3C). E2 did not significantly decrease the amplitude of CM tension at L_0_ (assessed at the initial cell length before the stretch, Figure 3D) or the slope of the active tension–length relationship (ATLR, Figure 3E). E2 reduced the slopes of the diastolic (passive) tension–length relationship (EDTLR) and end-systolic (total) tension–length relationship (ESTLR) by ~65% and ~59%, respectively (Figure 3F,G). FSG index (calculated as an ESTLR/EDTLR ratio) was not changed after the incubation with E2 (Figure 3H).

Thus, an E2-induced decrease in sarcomere shortening amplitude did not lead to a reduction in the auxotonic tension amplitude of ventricular CM. E2 decreased the ESTLR slope via a decrease in the EDTLR slope, indicating its impact on the passive properties of ventricular CM.

### 3.4. The Effects of 17β-Estradiol on the Myosin Function and Sarcomere Protein Phosphorylation

**Figure 4:**
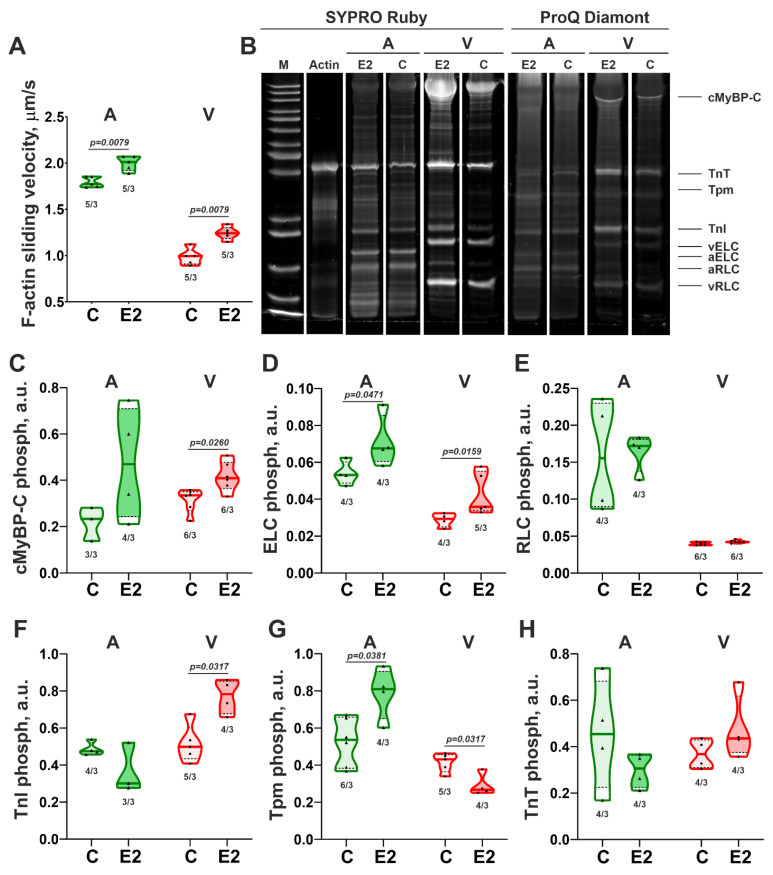
Effects of 17β-estradiol (E2) on sarcomeric contractile proteins, extracted from atrial and ventricular CM. (**A**) Sliding velocity of F-actin over myosin in the in vitro motility assay; (**B**) Representative gel stained with Pro-Q Diamond and SYPRO Ruby from the control group (C) and after 15 min incubation with E2: M—marker of molecular weight, cMyBP-C—cardiac myosin-binding protein C, TnT—troponin T, Tpm—tropomyosin, TnI—troponin I, aELC and vELC—atrial and ventricular essential light chain of myosin; aRLC and vRLC—atrial and ventricular myosin regulatory light chain; (**C**) cMyBP-C phosphorylation; (**D**) ELC phosphorylation; (**E**) RLC phosphorylation; (**F**) TnI phosphorylation; (**G**) Tpm phosphorylation; (**H**) TnT phosphorylation. A—atrial CM; V—ventricular CM. Data are presented as violin plots: bold line shows median, dashed lines indicate an Q1–Q3 interval. n/N—number of measurements from N hearts. The Mann–Whitney U-test is used to compare C and E2 groups.

To reveal molecular mechanisms responsible for E2 effects on CM contractility, the changes in the myosin function and phosphorylation levels of the main contractile proteins were assessed. E2 treatment induced an increase in the sliding velocity of F-actin over myosin from the atrial and ventricular CM by ~14% and ~25%, respectively (Figure 4A). Phosphorylation levels of contractile proteins after E2 incubation changed in a chamber-specific manner. Representative gels of phosphorylated proteins are shown in Figure 4B and Figure S1 (Supplementary Materials). In ventricular CM, E2 increased phosphorylation of cMyBP-C by ~22%, ELC by ~22%, and TnI by ~57% (Figure 4C,D,F) and did not affect RLC and TnT phosphorylation (Figure 4E,H). In atrial CM, the phosphorylation levels of cMyBP-C, RLC, TnI, and TnT did not change in response to E2 treatment, while the phosphorylation level of ELC increased by ~27%. Tpm phosphorylation decreased by ~38% in ventricular CM and increased by ~51% in atrial CM (Figure 4G).

These data suggest that E2 affects the myosin function, and phosphorylation of sarcomeric proteins may underlie the E2 induced changes in SL dynamics and tension-length relationship in CM, as will be discussed below.

## 4. Discussion

E2 exerts pleiotropic actions on the heart, influencing cardiac metabolism and regeneration and modulating the myocardial structure and electrical and contractile functions of the heart [28]. A number of studies have shown that E2 reveals inotropic effects on the human myocardium. Using preparations from the human left ventricular and right atrial myocardium, Sitzler and co-authors found that E2 concentrations greater than 30 μM have a negative inotropic effect [20]. Additionally, it was shown that 100 nM and 1 μM E2 suppresses the contractility of the rabbit heart [49]. E2 displays region-specific effects within the heart. Bening and co-authors found that low E2 levels have different effects on the maximum isometric force of the left and right human atrial appendage preparations [50]. In our previous work on ovariectomized rats, we found that deficiency of E2 had a negative inotropic response in single rat ventricular CM but not in atrial CM [32].

Because of the multiple and complex effects of E2 on the myocardial cells, we investigated the E2 influence on the myocardial contractility using isolated CM without the influence of other cell types, such as endothelial cells, fibroblasts, or immune cells. This approach allowed a more focused examination of CM-specific responses and mechanisms. For the first time, we established a direct effect of E2 on atrial and ventricular contractility at the single-cell and molecular levels, studying the amplitude and time-course characteristics of sarcomere shortening, force, and [Ca^2+^]_i_ transients in single cardiomyocytes, actin–myosin interaction, and phosphorylation levels of sarcomere proteins.

The main points of our study are as follows: (i) E2 regulates CM contractile function in a chamber-specific manner. We found that the contractile function of atrial CM was less sensitive to E2 treatment compared to ventricular CM. (ii) In single ventricular CM, E2 reduces the amplitude and maximum velocity of sarcomere shortening, as well as the slope of ESTLR (total tension–length relationship) through a decrease in the slope of EDTLR (passive tension–length relationship). (iii) In single atrial CM, E2 accelerates sarcomere shortening, reducing time-to-peak shortening. (iv) E2 induced acceleration of actin–myosin interaction in atrial and ventricular CM and increased phosphorylation of cMyBP-C and TnI in ventricular CM, which may contribute to the complex effects of E2 on the contractile function in ventricular CM.

### 4.1. E2 Effects on the Contraction of Atrial and Ventricular Cardiomyocytes

We found that the treatment of single isolated CM with E2 affects their contractile function. Previous studies demonstrated the direct E2 influence on the characteristics of CM contraction. Using ovariectomized rats, it has been shown that E2 deficiency reduces the amplitude and maximum velocity of shortening of single ventricular CM [32,51], and these changes are reversed by E2 treatment [51]. Here we show that in ventricular CM, 10 nM E2 decreased the amplitude and maximum velocity of sarcomere shortening pointing out that E2 directly affects sarcomere contractility in the ventricular myocardium. There is only one study on single ventricular CM that examined the E2 effects on APD, [Ca^2+^]_i_ transients, and CM contraction. Using CM from male guinea pigs, Jiang and co-authors [21] demonstrated that 10 and 30 µM E2 decreased cell shortening and APD duration, inhibiting L-type calcium current *I*_CaL_ and reducing systolic [Ca^2+^]_i_. In contrast, we did not find any effects of E2 on the characteristics of [Ca^2+^]_i_ transients that can be explained by the lower concentration of E2 used in our study.

Here we found that atrial CMs are less sensitive to E2 treatment than ventricular CMs. In atrial CMs, 10 nM E2 decreased the time-to-peak sarcomere shortening without changing the amplitude. Previously, Sitzler and co-authors [20] showed that E2 may induce the negative inotropic effect of atrial trabeculae at a higher E2 concentration (30 µM) compared to the ventricular trabeculae. We previously showed that E2 deficiency impaired the contractility in ventricular cardiomyocytes while no negative inotropic effects were seen in atrial cardiomyocytes [32].

The effects of E2 on the CM contraction may be associated with the changes in intracellular [Ca^2+^]_i_ or the function of sarcomere proteins. While the characteristics of [Ca^2+^]_i_ transients in CM were not altered, myosin properties and phosphorylation of sarcomere proteins changed after CM incubation with E2. Using the in vitro motility assay, we found that the sliding velocity of F-actin over atrial and ventricular myosin increased, pointing to an acceleration the of the cross-bridge cycle after E2 treatment. This result can be explained by an increase in the degree of ELC phosphorylation. Studies have shown that phosphorylation of the ELC affects myosin kinetics and myocardial force generation, especially under mechanical stress [52,53,54]. An acceleration of the cross-bridge cycle of myosin may explain a decrease in the time-to-peak sarcomere shortening observed in atrial CM. However, we found a reduction in the maximum velocity of sarcomere shortening in ventricular CM. This discrepancy can be explained by changes in post-translational modifications of other sarcomere proteins, including thin filament proteins.

In ventricular CM, which showed slowing of shortening, we found that E2 increased the phosphorylation levels of TnI and cMyBP-C. Previous studies have shown that phosphorylation of cMyBP-C and TnI via CaMKII or PKA, which are both controlled by E2 [10,55,56], may act coordinately to change the velocities of ventricular contraction and relaxation [57]. We hypothesize that E2-induced modulation of sarcomere shortening in ventricular CM is mediated by altered thin filament function and phosphorylation of regulatory proteins, rather than by changes in myosin properties.

E2 acts through estrogen receptors alpha (ERα), located in the nucleus, cytosol, and various membranes; estrogen receptors beta (ERβ), predominantly located in the nucleus and cytosol [58,59,60]; and membrane-bound receptor GPR30 (or GPER1) [61]. ERα and ERβ are involved in the genomic and non-genomic actions of E2 [62,63,64,65], and GPR30 mediates rapid non-genomic actions [61]. Previous studies have shown that ERα expression is higher in the ventricles compared to the atria [59,66]. The effects of GPR30 were described for both atrial and ventricular tissue [67,68], but there is no information about the atrial vs. ventricular differences in the GPR30 expression. We may suggest that the differences in the estrogen receptor expression and associated signaling pathways between atria and ventricles may lead to less sensitivity of atrial CM to E2 treatment compared to ventricular CM.

### 4.2. E2 Effects on Length-Dependent Force Production in Atrial and Ventricular Cardiomyocytes

In ventricular CM mechanically loaded by carbon fibers, we found that E2 did not significantly influence the tension amplitude before the application of the stretch, and it did not change the active tension–length relationship. Although the primary mechanism of force production in CM is sarcomere shortening, the cell viscosity, which is largely dependent on the cytoskeleton, also plays a significant role. The cell viscosity contributes to the resistance to mechanical loading during contraction, thereby affecting the overall force generated by CM [69]. The contribution of cellular viscosity may explain the inconsistency of E2-induced reduction of sarcomere shortening amplitude in mechanically non-loaded CM and non-altered tension amplitude in mechanically loaded CM. cMyBP-C phosphorylation modulates myofilament Ca^2+^ sensitivity in a length-dependent manner, contributing to the length-dependent activation of force production in CM [70]. We suggest that an increased level of cMyBP-C phosphorylation may maintain the active tension–length relationship in ventricular CM after E2 treatment.

We found that E2 treatment reduces the slope of the passive tension–length relationship, pointing to a decrease in the CM stiffness. This decrease might be associated with the changes in the properties of the cytoskeleton and/or titin. Data on the effects of estrogen on titin isoform expression and phosphorylation are very limited and require further research. Using ovariectomized rats, Kalász and co-authors did not find changes in passive force or titin phosphorylation in the LV [9]. Bupha-Intr and co-authors also found no differences in the passive force of multicellular myocardial preparations or cardiac titin isoforms between ovariectomized and sham rats, but, surprisingly, the increased myocardial stiffness in diabetic ovariectomized rats was accompanied by a shift toward a more compliant N2BA of cardiac titin isoforms compared to diabetic-sham rats [71]. In addition, estrogen may influence cytoskeletal function. Recent studies have shown that the estrogen metabolite, 2-methoxyestradiol, disrupts the microtubular network, reducing polymerized tubulin [72], which determines the viscoelastic properties of the myocardium [73,74]. The changes in the phosphorylation of cMyBP-C may also contribute to the altered myocardial stiffness. Rosas and co-authors showed that the slope of the pressure–volume relationship was decreased in mice expressing phosphorylation-deficient cMyBP-C and increased in mice expressing phosphomimetic cMyBP-C [75]. An increase in the phosphorylation of cMyBP-C observed in our study may contribute to a decrease in the ventricular CM stiffness.

## 5. Conclusions

This study demonstrates that E2 induces the direct chamber-specific contractile response of the myocardium at the single-cell and molecular levels. In ventricular CM, 10 nM E2 reduces the amplitude and maximum velocity of sarcomere shortening via an increase in TnI and cMyBP-C phosphorylation and decreases the stiffness of CM via increasing cMyBP-C phosphorylation. In atrial CM, E2 accelerates sarcomere shortening by accelerating cross-bridge cycling due to an increase in ELC phosphorylation. The chamber-sensitivity of myocardial contractile function to E2 should be considered for the potential side effects of HRT.

## 6. Limitations

We acknowledge the methodological limitations of this study. In vivo E2 treatment can be complex and may cause some inconsistencies among the acute E2 effects obtained in this study. While animal models provide valuable insights, direct extrapolation to human physiology should be done cautiously, and further studies in human subjects are necessary to confirm these observations. Due to the small volume of material available (single CM suspensions), we investigated the effect of E2 only on the functional properties of myosin and main sarcomeric protein phosphorylation. Further studies of thin filament properties are needed to provide a more comprehensive understanding of E2 effects on the entire contractile apparatus. When using a non-ratiometric dye for [Ca^2+^]_i_ transient measurements, signals had to be normalized to the resting fluorescence, making it challenging to study diastolic [Ca^2+^]_i_ levels. The analysis of the absolute diastolic [Ca^2+^]_i_ is needed for understanding myocardial stiffness properties. For the carbon fiber technique, we are limited by the studies of mechanically loaded ventricular CM because of the challenges of fixing and stretching thin and narrow atrial CM. The investigation of tension-length dependence in single atrial CM remains challenging and requires further technical innovations and targeted research in this area.

## Data Availability

All experimental data generated or analyzed during this study are included in this article.

## Supplementary Materials

The following supporting information can be downloaded at https://www.mdpi.com/article/10.3390/cells14080561/s1: Figure S1. Effects of 17β-estradiol (E2) on the absolute amplitude of sarcomere shortening in single atrial (A) and ventricular (V) CM. Figure S2. Examples of replicate gels for determining the degree of protein phosphorylation.

## References

[1] Meinhardt U., Mullis P.E. (2002). The essential role of the aromatase/p450arom. Seminars in Reproductive Medicine.

[2] Iorga A., Li J., Sharma S., Umar S., Bopassa J.C., Nadadur R.D., Centala A., Ren S., Saito T., Toro L. (2016). Rescue of pressure overload-induced heart failure by estrogen therapy. J. Am. Heart Assoc..

[3] Zhang S., Ma J., Wang X., Zhao D., Zhang J., Jiang L., Duan W., Wang X., Hong Z., Li Z. (2023). GPR30 alleviates pressure overload-induced myocardial hypertrophy in ovariectomized mice by regulating autophagy. Int. J. Mol. Sci..

[4] Rattanasopa C., Kirk J.A., Bupha-Intr T., Papadaki M., De Tombe P.P., Wattanapermpool J. (2019). Estrogen but not testosterone preserves myofilament function from doxorubicin-induced cardiotoxicity by reducing oxidative modifications. Am. J. Physiol.-Heart Circ. Physiol..

[5] Zhao Z., Wang H., Jessup J.A., Lindsey S.H., Chappell M.C., Groban L. (2014). Role of estrogen in diastolic dysfunction. Am. J. Physiol.-Heart Circ. Physiol..

[6] Dworatzek E., Mahmoodzadeh S., Schriever C., Kusumoto K., Kramer L., Santos G., Fliegner D., Leung Y.K., Ho S.M., Zimmermann W.H. (2019). Sex-specific regulation of collagen I and III expression by 17β-Estradiol in cardiac fibroblasts: Role of estrogen receptors. Cardiovasc. Res..

[7] Nuedling S., Kahlert S., Loebbert K., Doevendans P.A., Meyer R., Vetter H., Grohé C. (1999). 17β-Estradiol stimulates expression of endothelial and inducible NO synthase in rat myocardium in-vitro and in-vivo. Cardiovasc. Res..

[8] Donaldson C., Eder S., Baker C., Aronovitz M.J., Weiss A.D., Hall-Porter M., Wang F., Ackerman A., Karas R.H., Molkentin J.D. (2009). Estrogen attenuates left ventricular and cardiomyocyte hypertrophy by an estrogen receptor–dependent pathway that increases calcineurin degradation. Circ. Res..

[9] Kalász J., Pásztor Tóth E., Bodi B., Fagyas M., Toth A., Harjit Pal B., Vári S.G., Balog M., Blažetić S., Heffer M. (2014). Single acute stress-induced progesterone and ovariectomy alter cardiomyocyte contractile function in female rats. Croat. Med. J..

[10] Turdi S., Huff A.F., Pang J., He E.Y., Chen X., Wang S., Chen Y., Zhang Y., Ren J. (2015). 17-β estradiol attenuates ovariectomy-induced changes in cardiomyocyte contractile function via activation of AMP-activated protein kinase. Toxicol. Lett..

[11] Iorga A., Cunningham C.M., Moazeni S., Ruffenach G., Umar S., Eghbali M. (2017). The protective role of estrogen and estrogen receptors in cardiovascular disease and the controversial use of estrogen therapy. Biol. Sex Differ..

[12] Parks R.J., Bogachev O., Mackasey M., Ray G., Rose R.A., Howlett S.E. (2017). The impact of ovariectomy on cardiac excitation-contraction coupling is mediated through cAMP/PKA-dependent mechanisms. J. Mol. Cell. Cardiol..

[13] Yang H.Y., Firth J.M., Francis A.J., Alvarez-Laviada A., MacLeod K.T. (2017). Effect of ovariectomy on intracellular Ca^2+^ regulation in guinea pig cardiomyocytes. Am. J. Physiol.-Heart Circ. Physiol..

[14] Qian C., Liu J., Liu H. (2024). Targeting estrogen receptor signaling for treating heart failure. Heart Fail. Rev..

[15] Maluleke T.T., Millen A.M., Michel F.S. (2022). The effects of estrogen deficiency and aging on myocardial deformation and motion in normotensive female rats. Menopause.

[16] Bell J.R., Bernasochi G.B., Wollermann A.C., Raaijmakers A.J., Boon W.C., Simpson E.R., Curl C.L., Mellor K.M., Delbridge L.M. (2015). Myocardial and cardiomyocyte stress resilience is enhanced in aromatase-deficient female mouse hearts through CaMKIIδ activation. Endocrinology.

[17] Ramírez-Hernández D., López-Sanchez P., Lezama-Martínez D., Pérez-García E., Montoya-Hernández M.F.S., Aranda-Fraustro A., Flores-Monroy J. (2024). Early estrogen replacement therapy attenuates cardiac dysfunction caused by aging and ovariectomy in female Wistar rats. Front. Biosci. Landmark.

[18] Sobrano Fais R., Hoffer C., Moreno Vinasco L., Walts A., Cook T., Fisher A., Frump A.L., Woulfe K., Lahm T. (2023). NLRP3 Activation and Calcium-Dependent Contractile Function in Rat Right Ventricle Cardiomyocytes (RVCMs) Are Sexually Dimorphic and Controlled by 17β-Estradiol-via Estrogen Receptor-α. Circulation.

[19] van Eickels M., Patten R.D., Aronovitz M.J., Alsheikh-Ali A., Gostyla K., Celestin F., Grohe C., Mendelsohn M.E., Karas R.H. (2003). 17-beta-estradiol increases cardiac remodeling and mortality in mice with myocardial infarction. J. Am. Coll. Cardiol..

[20] Sitzler G., Lenz O., Kilter H., Rosee K.L. (1996). Investigation of the negative inotropic effects of 17β-oestradiol in human isolated myocardial tissues. Br. J. Pharmacol..

[21] Jiang C., Poole-Wilson P.A., Sarrel P.M., Mochizuki S., Collins P., MacLeod K.T. (1992). Effect of 17β-oestradiol on contraction, Ca^2+^ current and intracellular free Ca^2+^ in guinea-pig isolated cardiac myocytes. Br. J. Pharmacol..

[22] Ullrich N.D., Krust A., Collins P., MacLeod K.T. (2008). Genomic deletion of estrogen receptors ERα and ERβ does not alter estrogen-mediated inhibition of Ca^2+^ influx and contraction in murine cardiomyocytes. Am. J. Physiol.-Heart Circ. Physiol..

[23] Patten R.D., Pourati I., Aronovitz M.J., Alsheikh-Ali A., Eder S., Force T., Mendelsohn M.E., Karas R.H. (2008). 17 Beta-estradiol differentially affects left ventricular and cardiomyocyte hypertrophy following myocardial infarction and pressure overload. J. Card. Fail..

[24] Bell J.R., Bernasochi G.B., Varma U., Boon W.C., Ellem S.J., Risbridger G.P., Delbridge L.M. (2014). Aromatase transgenic upregulation modulates basal cardiac performance and the response to ischemic stress in male mice. Am. J. Physiol. Heart Circ. Physiol..

[25] Kurokawa J., Tamagawa M., Harada N., Honda S.I., Bai C.X., Nakaya H., Furukawa T. (2008). Acute effects of oestrogen on the guinea pig and human I_Kr_ channels and drug-induced prolongation of cardiac repolarization. J. Physiol..

[26] Anderson S.E., Kirkland D.M., Beyschau A., Cala P.M. (2005). Acute effects of 17β-estradiol on myocardial pH, Na^+^, and Ca^2+^ and ischemia-reperfusion injury. Am. J. Physiol. Cell Physiol..

[27] Ohya S., Kuwata Y., Sakamoto K., Muraki K., Imaizumi Y. (2005). Cardioprotective effects of estradiol include the activation of large-conductance Ca^2+^-activated K^+^ channels in cardiac mitochondria. Am. J. Physiol. Heart Circ. Physiol..

[28] Luo T., Kim J.K. (2016). The role of estrogen and estrogen receptors on cardiomyocytes: An overview. Can. J. Cardiol..

[29] Lee J., Kim Y., Park H., Kim C., Cho S., Kim J. (2021). Clinical impact of hormone replacement therapy on atrial fibrillation in postmenopausal women: A nationwide cohort study. J. Clin. Med..

[30] Wells S.P., O’Shea C., Hayes S., Weeks K.L., Kirchhof P., Delbridge L.M., Pavlovic D., Bell J.R. (2024). Male and female atria exhibit distinct acute electrophysiological responses to sex steroids. J. Mol. Cell. Cardiol. Plus.

[31] Bernasochi G.B., Boon W.C., Curl C.L., Varma U., Pepe S., Tare M., Bell J.R. (2017). Pericardial adipose and aromatase: A new translational target for aging, obesity and arrhythmogenesis?. J. Mol. Cell. Cardiol..

[32] Khokhlova A., Myachina T., Butova X., Volzhaninov D., Berg V., Kochurova A., Kuznetsov D., Mukhlynina E., Kopylova G., Shchepkin D. (2021). Differing effects of estrogen deficiency on the contractile function of atrial and ventricular myocardium. Biochem. Biophys. Res. Commun..

[33] Jankowski M., Rachelska G., Donghao W., McCann S.M., Gutkowska J. (2001). Estrogen receptors activate atrial natriuretic peptide in the rat heart. Proc. Natl. Acad. Sci. USA.

[34] Qiao G.F., Li B.Y., Lu Y.J., Fu Y.L., Schild J.H. (2009). 17β-estradiol restores excitability of a sexually dimorphic subset of myelinated vagal afferents in ovariectomized rats. Am. J. Physiol. Cell Physiol..

[35] Parini P., Angelin B., Stavréus-Evers A., Freyschuss B., Eriksson H., Rudling M. (2000). Biphasic effects of the natural estrogen 17beta-estradiol on hepatic cholesterol metabolism in intact female rats. Arterioscler. Thromb. Vasc. Biol..

[36] Pelzer T., Schumann M., Neumann M., deJager T., Stimpel M., Serfling E., Neyses L. (2000). 17β-estradiol prevents programmed cell death in cardiac myocytes. Biochem. Biophys. Res. Commun..

[37] Al-Rubaiee M., Gangula P.R., Millis R.M., Walker R.K., Umoh N.A., Cousins V.M., Jeffress M.A., Haddad G.E. (2013). Inotropic and lusitropic effects of calcitonin gene-related peptide in the heart. Am. J. Physiol. -Heart Circ. Physiol..

[38] Butova X.A., Myachina T.A., Khokhlova A.D. (2021). A combined Langendorff-injection technique for simultaneous isolation of single cardiomyocytes from atria and ventricles of the rat heart. MethodsX.

[39] Khokhlova A., Myachina T., Butova X., Kochurova A., Polyakova E., Galagudza M., Solovyova O., Kopylova G., Shchepkin D. (2022). The acute effects of leptin on the contractility of isolated rat atrial and ventricular cardiomyocytes. Int. J. Mol. Sci..

[40] Myachina T.A., Butova K.A., Lookin O.N. (2019). Development and program implementation of an algorithm to estimate the mean sarcomere length of a cardiomyocyte. Biophysics.

[41] Khokhlova A., Konovalov P., Iribe G., Solovyova O., Katsnelson L. (2020). The effects of mechanical preload on transmural differences in mechano-calcium-electric feedback in single cardiomyocytes: Experiments and mathematical models. Front. Physiol..

[42] Volzhaninov D., Khokhlova A. Parallel Control of Two Digital Micromanipulators for Biomechanical Experiments Using LabVIEW. Proceedings of the 2019 Ural Symposium on Biomedical Engineering, Radioelectronics and Information Technology (USBEREIT).

[43] Nishimura S., Yasuda S.I., Katoh M., Yamada K.P., Yamashita H., Saeki Y., Sunagawa K., Nagai R., Hisada T., Sugiura S. (2004). Single cell mechanics of rat cardiomyocytes under isometric, unloaded, and physiologically loaded conditions. Am. J. Physiol. Heart Circ. Physiol..

[44] Bollensdorff C., Lookin O., Kohl P. (2011). Assessment of contractility in intact ventricular cardiomyocytes using the dimensionless ‘Frank–Starling Gain’index. Pflügers Arch. Eur. J. Physiol..

[45] Khokhlova A., Solovyova O., Kohl P., Peyronnet R. (2022). Single cardiomyocytes from papillary muscles show lower preload-dependent activation of force compared to cardiomyocytes from the left ventricular free wall. J. Mol. Cell. Cardiol..

[46] Margossian S.S., Lowey S. (1982). Preparation of myosin and its subfragments from rabbit skeletal muscle. Methods in Enzymology.

[47] Pardee J.D., Spudich A.J. (1982). Purification of muscle actin. Methods in Enzymology.

[48] Quinn T.A., Kohl P. (2021). Cardiac mechano-electric coupling: Acute effects of mechanical stimulation on heart rate and rhythm. Physiol. Rev..

[49] Raddino R., Manca C., Poli E., Bolognesi R., Visioli O. (1986). Effects of 17 beta-estradiol on the isolated rabbit heart. Arch. Int. Pharmacodyn. Ther..

[50] Bening C., Genser B., Keller D., Müller-Altrock S., Radakovic D., Penov K., Hassan M., Aleksic I., Leyh R., Madrahimov N. (2023). Impact of estradiol, testosterone and their ratio on left and right auricular myofilament function in male and female patients undergoing coronary artery bypass grafting. BMC Cardiovasc. Disord..

[51] Ren J., Hintz K.K., Roughead Z.K., Duan J., Colligan P.B., Ren B.H., Lee K.J., Zeng H. (2003). Impact of estrogen replacement on ventricular myocyte contractile function and protein kinase B/Akt activation. Am. J. Physiol. Heart Circ. Physiol..

[52] Müller M., Eghbalian R., Boeckel J.N., Frese K.S., Haas J., Kayvanpour E., Sedaghat-Hamedani F., Lackner M.K., Tugrul O.F., Ruppert T. (2022). NIMA-related kinase 9 regulates the phosphorylation of the essential myosin light chain in the heart. Nat. Commun..

[53] Scheid L.M., Mosqueira M., Hein S., Kossack M., Juergensen L., Mueller M., Meder B., Fink R.H., Katus H.A., Hassel D. (2016). Essential light chain S195 phosphorylation is required for cardiac adaptation under physical stress. Cardiovasc. Res..

[54] Meder B., Laufer C., Hassel D., Just S., Marquart S., Vogel B., Hess A., Fishman M.C., Katus H.A., Rottbauer W. (2009). A single serine in the carboxyl terminus of cardiac essential myosin light chain-1 controls cardiomyocyte contractility in vivo. Circ. Res..

[55] Ma Y., Cheng W.T., Wu S., Wong T.M. (2009). Oestrogen confers cardioprotection by suppressing Ca^2+^/calmodulin-dependent protein kinase II. Br. J. Pharmacol..

[56] Mahmoodzadeh S., Dworatzek E. (2019). The role of 17β-estradiol and estrogen receptors in regulation of Ca2+ channels and mitochondrial function in cardiomyocytes. Front. Endocrinol..

[57] Tong C.W., Gaffin R.D., Zawieja D.C., Muthuchamy M. (2004). Roles of phosphorylation of myosin binding protein-C and troponin I in mouse cardiac muscle twitch dynamics. J. Physiol..

[58] Ropero A.B., Eghbali M., Minosyan T.Y., Tang G., Toro L., Stefani E. (2006). Heart estrogen receptor alpha: Distinct membrane and nuclear distribution patterns and regulation by estrogen. J. Mol. Cell. Cardiol..

[59] Lizotte E., Grandy S.A., Tremblay A., Allen B.G., Fiset C. (2009). Expression, distribution and regulation of sex steroid hormone receptors in mouse heart. Cell. Physiol. Biochem..

[60] Iorga A., Umar S., Ruffenach G., Aryan L., Li J., Sharma S., Motayagheni N., Nadadur R.D., Bopassa J.C., Eghbali M. (2018). Estrogen rescues heart failure through estrogen receptor Beta activation. Biol. Sex Differ..

[61] Machuki J.O., Zhang H.Y., Harding S.E., Sun H. (2018). Molecular pathways of oestrogen receptors and β-adrenergic receptors in cardiac cells: Recognition of their similarities, interactions and therapeutic value. Acta Physiol..

[62] Grohé C., Kahlert S., Löbbert K., Stimpel M., Karas R.H., Vetter H., Neyses L. (1997). Cardiac myocytes and fibroblasts contain functional estrogen receptors. FEBS Lett..

[63] Lipovka Y., Chen H., Vagner J., Price T.J., Tsao T.S., Konhilas J.P. (2015). Oestrogen receptors interact with the α-catalytic subunit of AMP-activated protein kinase. Biosci. Rep..

[64] Huang P.C., Kuo W.W., Shen C.Y., Chen Y.F., Lin Y.M., Ho T.J., Padma V.V., Lo J.F., Huang C.Y., Huang C.Y. (2016). Anthocyanin attenuates doxorubicin-induced cardiomyotoxicity via estrogen receptor-α/β and stabilizes HSF1 to inhibit the IGF-IIR apoptotic pathway. Int. J. Mol. Sci..

[65] Schuster I., Mahmoodzadeh S., Dworatzek E., Jaisser F., Messaoudi S., Morano I., Regitz-Zagrosek V. (2016). Cardiomyocyte-specific overexpression of oestrogen receptor β improves survival and cardiac function after myocardial infarction in female and male mice. Clin. Sci..

[66] Pugach E.K., Blenck C.L., Dragavon J.M., Langer S.J., Leinwand L.A. (2016). Estrogen receptor profiling and activity in cardiac myocytes. Mol. Cell. Endocrinol..

[67] Weil B.R., Manukyan M.C., Herrmann J.L., Wang Y., Abarbanell A.M., Poynter J.A., Meldrum D.R. (2010). Signaling via GPR30 protects the myocardium from ischemia/reperfusion injury. Surgery.

[68] Liu D., Zhan Y., Ono K., Yin Y., Wang L., Wei M., Ji L., Liu M., Liu G., Zhou X. (2022). Pharmacological activation of estrogenic receptor G protein-coupled receptor 30 attenuates angiotensin II-induced atrial fibrosis in ovariectomized mice by modulating TGF-β1/smad pathway. Mol. Biol. Rep..

[69] Caporizzo M.A., Chen C.Y., Salomon A.K., Margulies K.B., Prosser B.L. (2018). Microtubules provide a viscoelastic resistance to myocyte motion. Biophys. J..

[70] Mamidi R., Gresham K.S., Verma S., Stelzer J.E. (2016). Cardiac myosin binding protein-C phosphorylation modulates myofilament length-dependent activation. Front. Physiol..

[71] Bupha-Intr T., Oo Y.W., Wattanapermpool J. (2011). Increased myocardial stiffness with maintenance of length-dependent calcium activation by female sex hormones in diabetic rats. Am. J. Physiol.-Heart Circ. Physiol..

[72] Shen J.B., Pappano A.J. (2008). An estrogen metabolite, 2-methoxyestradiol, disrupts cardiac microtubules and unmasks muscarinic inhibition of calcium current. J. Pharmacol. Exp. Ther..

[73] Vite A., Caporizzo M.A., Corbin E.A., Brandimarto J., McAfee Q., Livingston C.E., Prosser B.L., Margulies K.B. (2022). Extracellular stiffness induces contractile dysfunction in adult cardiomyocytes via cell-autonomous and microtubule-dependent mechanisms. Basic Res. Cardiol..

[74] Caporizzo M.A., Prosser B.L. (2022). The microtubule cytoskeleton in cardiac mechanics and heart failure. Nat. Rev. Cardiol..

[75] Rosas P.C., Liu Y., Abdalla M.I., Thomas C.M., Kidwell D.T., Dusio G.F., Mukhopadhyay D., Kumar R., Baker K.M., Mitchell B.M. (2015). Phosphorylation of cardiac Myosin-binding protein-C is a critical mediator of diastolic function. Circ. Heart Fail..

