# Supplementary material for: The Contractile Function of Ventricular Cardiomyocytes Is More Sensitive to Acute 17β-Estradiol Treatment Compared to Atrial Cardiomyocytes
Source: Cells. 2025 Apr 8;14(8):561. doi: 10.3390/cells14080561 (PMC12026394; doi:10.3390/cells14080561)
Supplement: Supplementary file 1 [file cells-14-00561-s001.zip › cells-3555210-supplementary.pdf]

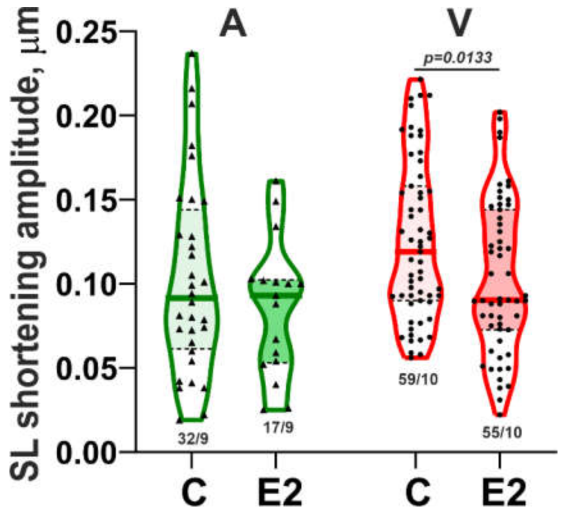

Figure S1. Direct effect of 17β-estradiol (E2) on absolute amplitude of sarcomere shortening in single atrial (A) and ventricular (V) CM. Data are presented in violin plots: bold line shows median, dashed lines indicate an Q1–Q3 interval. Each dot represents an individual CM. Mann-Whitney U-test, $p < 0.05$.

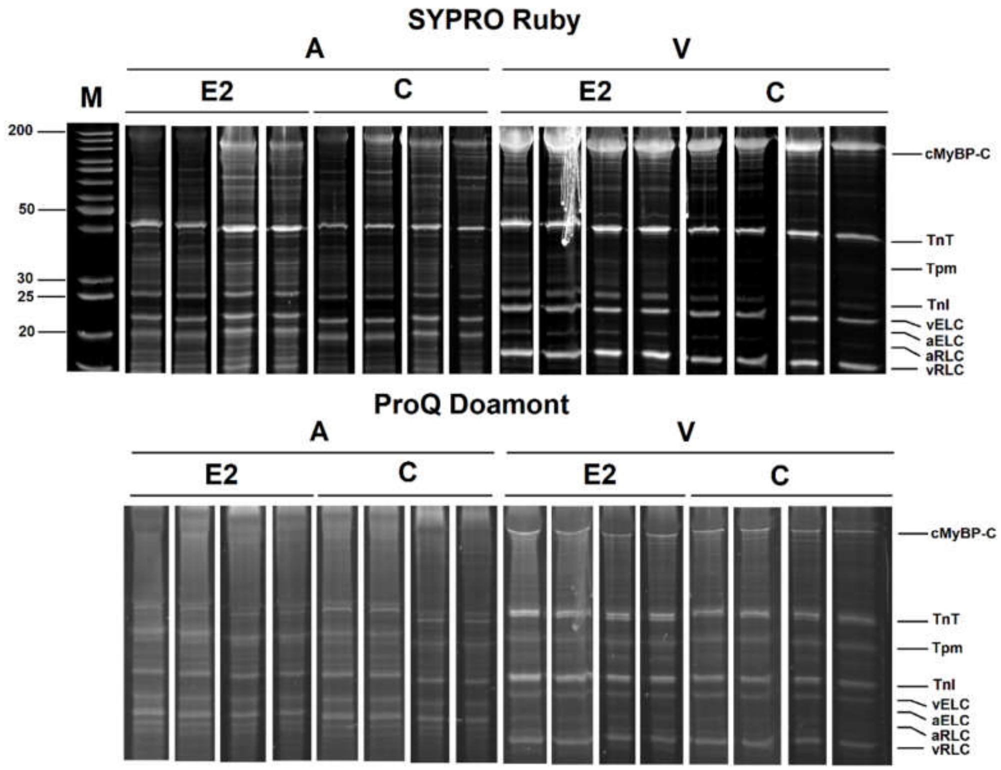

Figure S2. Examples of replicate gels for determining the degree of protein phosphorylation. Gel stained with Pro-Q Diamond and SYPRO Ruby from the control group (C) and after 15 min incubation with 10 nM E2: M – marker of molecular weight (Thermo Fisher Scientific, USA), cMyBP-C – cardiac myosin-binding protein C, TnT – troponin T, Tpm – tropomyosin, TnI – troponin I, aELC and vELC – atrial and ventricular essential light chain of myosin; aRLC and vRLC – atrial and ventricular myosin regulatory light chain.
